# Predictive factors and risk model for depression in patients with type 2 diabetes mellitus: a comprehensive analysis of comorbidities and clinical indicators

**DOI:** 10.3389/fendo.2025.1555142

**Published:** 2025-03-05

**Authors:** Chengzheng Duan, Cheng Luo, Weifeng Jiang, Hui Xu, Yexing Chen, Shiyu Xu, Xiaofang Zhang, Xiaoli Chen, Dongjuan He

**Affiliations:** ^1^ The Quzhou Affiliated Hospital of Wenzhou Medical University, Quzhou People’s Hospital, Quzhou, China; ^2^ State Key Laboratory on Technologies for Chinese Medicine Pharmaceutical Process Control and Intelligent Manufacture, Nanjing University of Chinese Medicine, Nanjing, China

**Keywords:** type 2 diabetes, depression, comorbidity, cumulative illness rating scale, predictive model

## Abstract

**Objective:**

Depression is highly prevalent among individuals with type 2 diabetes mellitus (T2DM), often compounded by multiple chronic conditions. This study aimed to identify the key factors influencing depression in this population, with a particular focus on the relationship between the Cumulative Illness Rating Scale (CIRS) score and depression, and to evaluate the predictive value of a model incorporating sex, body mass index (BMI), low-density lipoprotein cholesterol (LDL-C), and CIRS score.

**Methods:**

A total of 308 hospitalized patients with type 2 diabetes from Quzhou Hospital, Wenzhou Medical University were enrolled. Their clinical and biochemical data were collected, alongside assessments of comorbidities and depressive symptoms using the CIRS and Self-Rating Depression Scale (SDS), respectively. LASSO regression with 10-fold cross-validation was used to identify the optimal variables for the predictive model. Multivariate analysis was performed to assess the independent associations between sex, BMI, LDL-C, and CIRS score with depression. The relationship between CIRS scores and depression was further explored across various subgroups. The predictive model’s value was assessed through ROC curve analysis.

**Results:**

Female sex (OR: 2.48, 95% CI: 1.50-4.10, p < 0.001), lower BMI (OR: 0.92, 95% CI: 0.86-0.98, p = 0.015), lower LDL-C (OR: 0.77, 95% CI: 0.61-0.98, p = 0.031), and higher CIRS scores (OR: 1.11, 95% CI: 1.05-1.18, p < 0.001) were independently linked to depression after adjusting for clinical variables. A strong association between CIRS score and depression was observed, particularly in males, patients under 60 years old, those with a disease duration of less than 5 years, and individuals with no history of smoking or alcohol consumption. Additionally, a predictive model incorporating sex, BMI, LDL-C, and CIRS score demonstrated high accuracy in identifying patients at risk for depression.

**Conclusions:**

Female, lower BMI, lower LDL-C and higher CIRS score were independently associated with depression in patients with type 2 diabetes. The CIRS score appeared to be more effective in predicting depression risk in people who were male, younger, shorter DM duration, no smoking or no drinking. A more comprehensive prediction model could help clinicians identify patients with type 2 diabetes who are at risk for depression.

## 1 Introduction

Diabetes mellitus (DM) is a chronic metabolic disorder that has reached epidemic proportions worldwide. The global prevalence of diabetes exceeds 500 million individuals, with projections indicating a staggering rise to 1.31 billion cases by 2050 (1). As one of the leading contributors to disability and mortality, diabetes represents a major public health challenge of the 21st century, as underscored by The Lancet (2). Beyond its direct physiological impact, diabetes significantly amplifies the burden of healthcare systems due to its association with an array of comorbid conditions.

Comorbidities, defined as the co-occurrence of two or more chronic diseases within the same individual, are particularly prevalent among patients with diabetes (3, 4). These conditions, which commonly include hypertension, cardiovascular disease, nephropathy, and depression, complicate disease management, adversely affect prognosis, and substantially reduce quality of life. Studies indicate that people with type 2 diabetes generally have multiple comorbidities, and at least 77% of them have more than one comorbidities (5). This high burden of comorbidities underscores the need for a more holistic approach to the management of diabetic patients, one that accounts for both physiological and psychological health. Among the various comorbidities associated with diabetes, depression has emerged as a particularly critical concern due to its bidirectional relationship with diabetes and its profound implications for patient outcomes (6–8). Depression is more prevalent in diabetic populations than in the general population, affecting nearly 23.2% of diabetic patients (9). The risk of depression is particularly pronounced among women, with studies consistently reporting higher prevalence rates in females compared to males (10). Prevalence rates among diabetic individuals vary widely, ranging from 3.8% to 49.5%, reflecting differences in population demographics, diagnostic criteria, and healthcare access (11). Depression in diabetic patients is associated with a cascade of adverse outcomes, including poor glycemic control (12), reduced adherence to treatment (13), diminished quality of life (14), and increased all-cause mortality (15). The interplay between diabetes and depression forms a self-perpetuating cycle, wherein the stress of managing diabetes exacerbates psychological distress, which in turn worsens metabolic control and accelerates diabetes-related complications (16). This complex relationship necessitates early identification and management of depression to mitigate its impact on diabetes progression and overall health outcomes.

Despite the growing recognition of depression as a critical comorbidity, its detection in diabetic patients remains challenging. Conventional screening methods often fail to capture the multifactorial nature of depression risk in this population. In this context, the Cumulative Illness Rating Scale (CIRS) has emerged as a promising tool. The CIRS provides a comprehensive assessment of comorbid burden by quantifying the severity of chronic illnesses across multiple organ systems (17). While extensively validated in geriatric populations and patients with multimorbidity, the utility of the CIRS in predicting mental health outcomes, particularly depression, in diabetic patients remains underexplored.

This study aims to fill this gap by investigating the relationship between CIRS scores and depression in patients with type 2 diabetes. Using a cohort of hospitalized diabetic patients, we examine the role of demographic factors (e.g., sex), clinical parameters (e.g., body mass index [BMI], low-density lipoprotein cholesterol [LDL-C] levels), and comorbid burden (CIRS scores) in predicting depression risk. By developing and validating a predictive model, we aim to equip clinicians with a practical tool for early identification of high-risk patients, thereby facilitating timely interventions. This approach not only addresses the immediate psychological needs of diabetic patients but also has the potential to improve their long-term physical and metabolic outcomes.

## 2 Methods and materials

### 2.1 Study design

**Figure 1:**
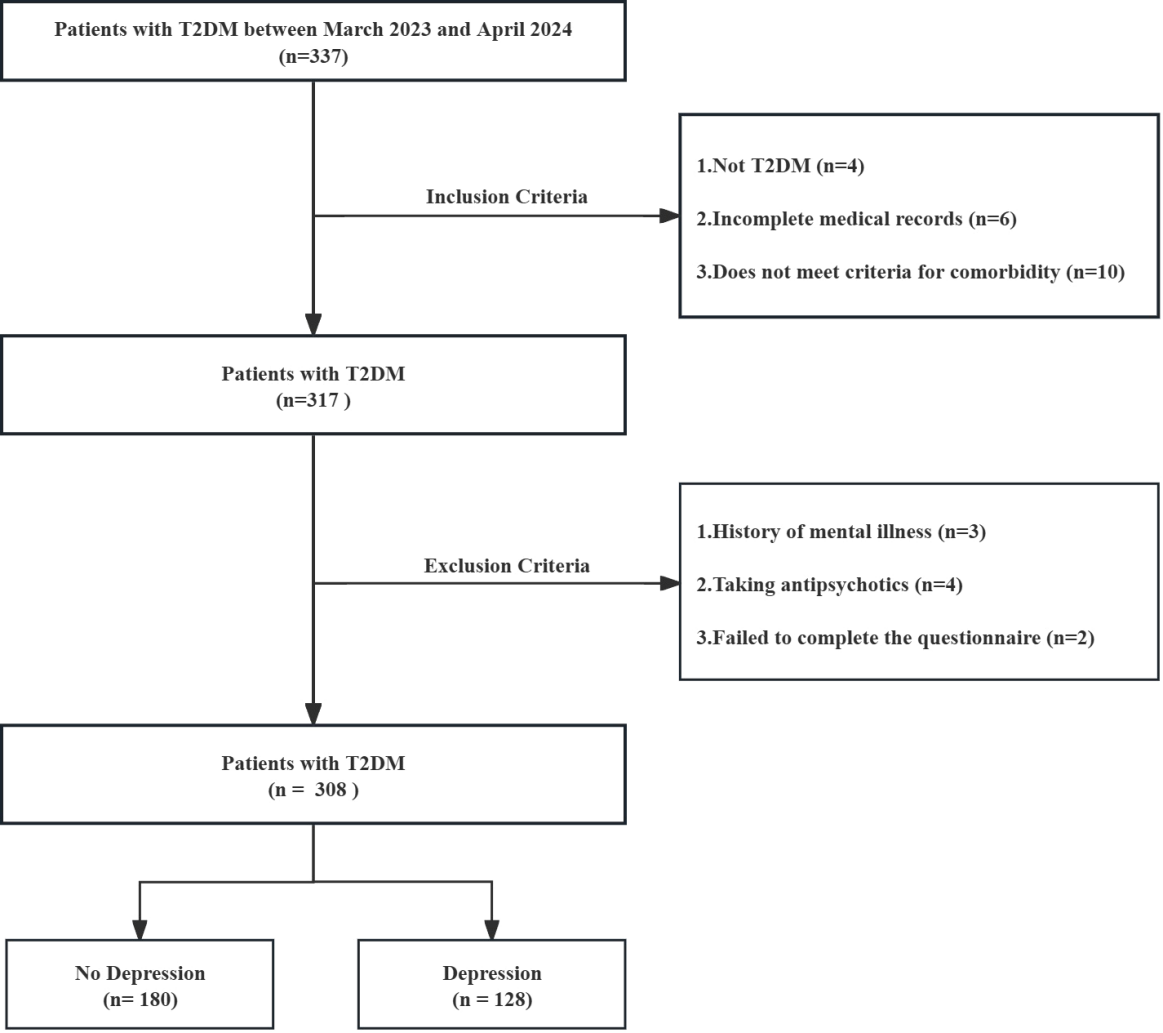
Flowchart for screening research subjects.

This study included 337 patients with T2DM hospitalized at Hospital, between March 2023 and April 2024. According to the inclusion and exclusion criteria, 308 patients with T2DM were screened. Inclusion criteria: (1) Adults (≥18 years old) with a confirmed diagnosis of T2DM based on standard diagnostic criteria; (2) Presence of at least one additional chronic condition lasting ≥ 6 months, such as hypertension or chronic obstructive pulmonary disease (COPD); (3) Ability to communicate effectively and cooperate to complete the required assessments; (4) Availability of comprehensive medical records. Exclusion criteria: (1) Current or past diagnosis of psychiatric disorders, or ongoing treatment with antipsychotic medications; (2) Positive family history of mental illness; (3) Withdrawal due to lack of cooperation or incomplete participation; (4) Insufficient clinical data for evaluation. The screening process is shown in **Figure 1**. Ethical approval was obtained from the institutional review board, and all participants provided written informed consent prior to inclusion.

### 2.2 Data collection

#### 2.2.1 Demographic and clinical information

Baseline data including age, sex, education level, smoking history and alcohol use, and body mass index (BMI) and duration of diabetes were collected. Biochemical parameters, such as fasting blood glucose (FPG), glycated hemoglobin (HbA1c), triglyceride (TG), total cholesterol (TC), high-density lipoprotein cholesterol (HDL-C), low-density lipoprotein cholesterol (LDL-C), and C-reactive protein (CRP), were recorded at admission.

#### 2.2.2 Psychological assessment

The psychological status of participants was assessed using the self-rating Depression Scale (SDS), developed by Zung (18). The SDS evaluates subjective experiences of depression using 20 items, scored on a four-point Likert scale. The depression index was calculated as the cumulative score divided by 80, with the following classifications: (1) No depression: < 0.50; (2) Mild depression: 0.50–0.59; (3) Moderate depression: 0.60-0.69; (4) Severe depression: ≥ 0.70.

#### 2.2.3 Comorbidity assessment

The CIRS provides a comprehensive assessment of comorbidities by evaluating the degree of damage across various organ systems. Unlike other scales that focus solely on current diseases, the CIRS also accounts for historical diseases, allowing for a more thorough evaluation of both present and past health conditions. The scale has been widely adopted due to its ability to predict clinical outcomes, including hospital readmission rates and both short-term and long-term mortality (19–22).

For this study, two specially trained researchers used the CIRS to assess the comorbidity status of patients. Detailed patient medical histories, including past medical records, were collected to ensure accuracy in scoring. The CIRS evaluates 14 organ systems, including heart, blood vessels, respiration, eye, ear, nose and throat, digestive system, reproductive system, bone and skin, and nervous system. Each system is scored based on disease severity, with 5 grades: (1) Grade 0, no damage (0 points); (2) Grade 1, mild damage (1 point); (3) Grade 2, moderate damage (2 points); (4) Grade 3, severe damage, with limited function and potential disability (3 points); (5) Grade 4, life-threatening damage (4 points). The total score ranges from 0 to 56, with higher scores indicating more severe comorbidity.

### 2.3 Statistical analysis

The statistical analyses were performed using R, a software environment (R version 3.3.2). Continuous variables were tested for normality using the Shapiro-Wilk test. Normally distributed data were presented as mean ± SD and compared using the independent-samples t-test. Non-normally distributed data were expressed as medians (interquartile range, IQR) and analyzed using the Mann-Whitney U test. The categorical variables were expressed as number and percentage and analyzed with the chi-square test. The best variables screened from the Lasso regression were included in the multivariate logistic regression for analysis, and a prediction model was constructed. To assess the predictive capability of prospective factors and model,receiver operating characteristic (ROC) analysis were conducted. In addition, patients were grouped according to different population characteristics, and the relationship between variables among different populations was analyzed by logistic regression. p < 0.05 was considered to be statistically significant.

## 3 Result

This study included 308 patients with T2DM, comprising 62.66% males (193/308) and 37.34% females (115/308). The median age of the patients was 60 years (IQR: 51, 70), and the median CIRS score was 10 (IQR: 7, 13). Among the cohort, 58.44% (180/308) exhibited no depression, while 41.56% (128/308) had depression (**Table 1**).

**Table 1:** The characteristics of the patients included in our study.

Variables	Overall (n = 308)	No Depression(n = 180)	Depression (n = 128)	*p*-value
Age (years)^a^	60 (51, 70)	57 (49.75, 66.25)	63(52.75, 74.00)	0.001*
Sex (%)^b^				<0.001*
male	193 (62.66)	127 (70.56)	66 (51.56)	
female	115 (37.34)	53 (29.44)	62 (48.44)	
BMI (kg/m^2^)^a^	24.41 (22.49, 26.58)	24.41 (22.86, 27.08)	24.41 (22.04, 26.07)	0.046*
DM duration (years)^b^				0.034*
<5	135 (43.83)	88 (48.89)	47 (36.72)	
≥5	173 (56.17)	92 (51.11)	81 (63.28)	
Educational level (%)^b^				0.011*
illiterate	43 (13.96)	16 (8.89)	27 (21.09)	
primary school	78 (25.32)	43 (23.89)	35 (27.34)	
middle school	97 (31.49)	60 (33.33)	37 (28.91)	
high school	42 (13.64)	26 (14.44)	16 (12.50)	
college and above	48 (15.58)	35 (19.44)	13 (10.16)	
Smoking (%)^b^				0.006*
No	174 (56.49)	90 (50.00)	84 (65.62)	
Yes	134 (43.51)	90 (50.00)	44 (34.38)	
Drinking (%)^b^				0.086
No	197 (63.96)	108 (60.00)	89 (69.53)	
Yes	111 (36.04)	72 (40.00)	39 (30.47)	
HBA1c (%)^a^	8.39 (7.00, 10.62)	8.62 (7.11, 10.95)	8.02 (6.90, 10.12)	0.092
FPG (mmol/L)^a^	7.40 (6.20, 8.88)	7.30 (6.10, 8.80)	7.60 (6.41, 8.90)	0.542
PPG (mmol/L)^a^	11.65 (9.60, 15.05)	11.60 (9.38, 15.58)	11.70 (9.73, 13.80)	0.616
AST (U/L)^a^	18.50 (14.20, 26.70)	18.90 (14.50, 25.40)	18.05 (14.20, 27.50)	0.881
ALT (U/L)^a^	19.65 (13.40, 32.28)	20.85 (14.70, 34.62)	17.25 (12.70, 28.08)	0.011*
TG (mmol/L)^a^	1.64 (1.19, 2.50)	1.73 (1.21, 2.65)	1.61 (1.17, 2.28)	0.258
TC (mmol/L)^a^	4.39 (3.58, 5.25)	4.51 (3.76, 5.50)	4.12 (3.39, 4.95)	0.002*
LDL-C (mmol/L)^a^	2.56 (1.87, 3.30)	2.65 (2.05, 3.48)	2.44 (1.72, 3.13)	0.014*
HDL-C (mmol/L)^a^	1.07 (0.90, 1.25)	1.07 (0.92, 1.27)	1.07 (0.88, 1.20)	0.196
RC (mg/dL)^a^	0.51 (0.32, 0.80)	0.51 (0.31, 0.84)	0.52 (0.38, 0.75)	0.832
UA (μmol/L)^a^	317.70(259.45, 396.88)	312.60(259.85, 382.20)	324.50(258.05, 425.22)	0.201
HCY (mol/L)^a^	11.85 (9.38, 15.68)	11.66 (9.26, 14.93)	12.25 (9.68, 17.06)	0.120
CRP (mg/L)^a^	2.08 (0.83, 5.67)	2.01 (0.83, 4.73)	2.50 (0.82, 7.18)	0.276
CIRS score ^a^	10 (7, 13)	9 (6, 12)	11 (8, 15)	<0.001*

HbA1c, glycated hemoglobin; FPG, fasting plasma glucose; PPG, postprandial plasma glucose; AST, aspartate aminotransferase; ALT, alanine aminotransferase; TG, triglyceride; TC, total cholesterol; LDL-C, low-density lipoprotein cholesterol; HDL-C, high-density lipoprotein cholesterol; RC, remnant cholesterol; UA, urine acid; HCY, homocysteine; CRP, C-reactive protein.^a^Median (1st Quartile, 3st Quartile) was used for continuous, non-normal distributed variables; ^b^The categorical variables were expressed as number and percentage *p < 0.05.

**Figure 2:**
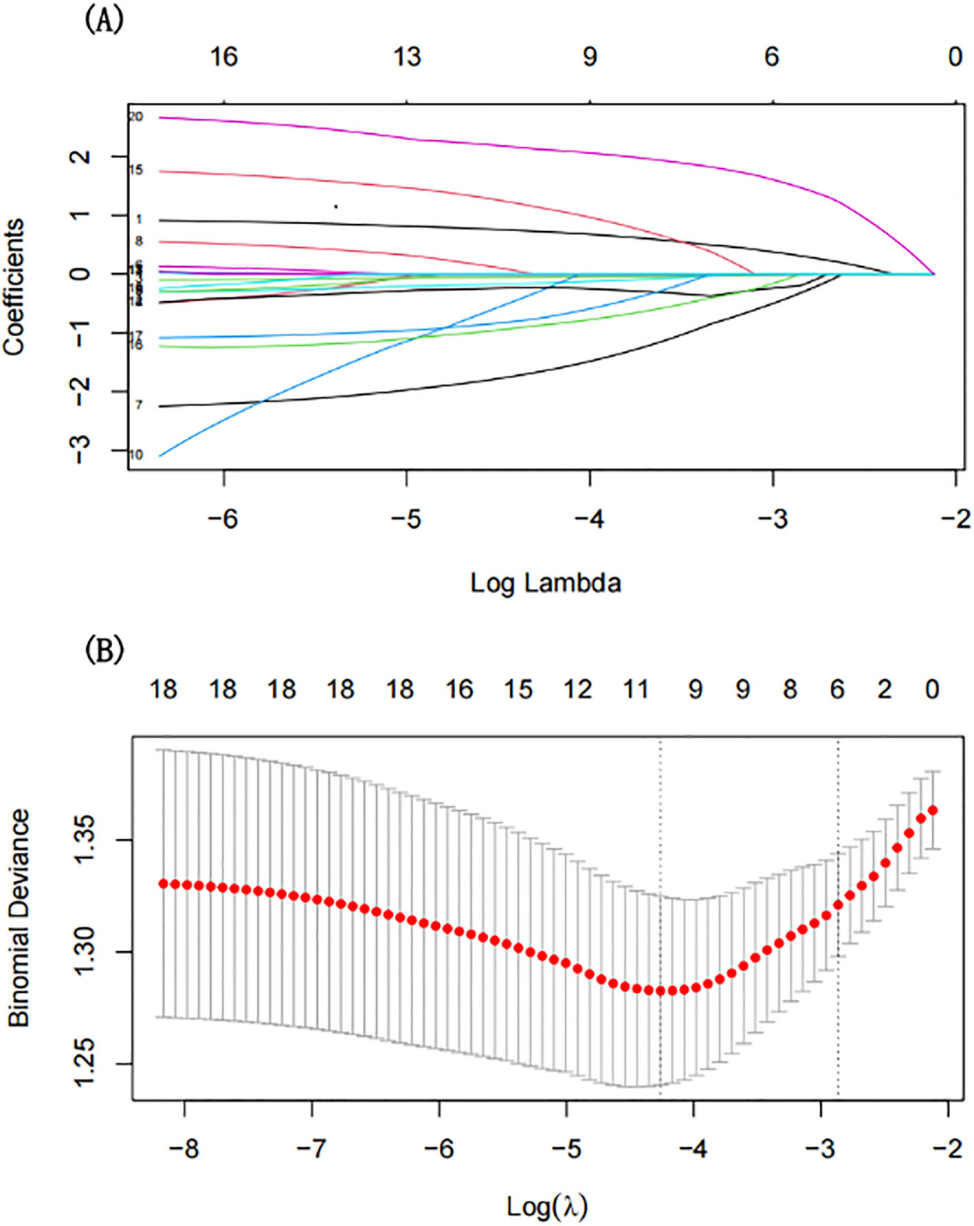
Predictor selection using LASSO regression. **(A)** The coefficient profile generated for non-zero coefficients based on the logarithmic (lambda) sequence. **(B)** The optimal lambda value selected via tenfold cross-validation. The partial likelihood deviation (binomial deviation) curve relative to log (lambda) was plotted. A virtual vertical line at the optimal value was drawn using one SE of minimum criterion (the 1-SE criterion).

To understand the factors that may contribute to depression in people with T2DM, we use LASSO regression, which can help us to reduce the risk of overfitting by eliminating irrelevant or redundant variables when there are too many of them. This method helps us identify the most important variables that could be linked to depression. Our analysis highlighted several key factors: sex, educational level, BMI, TC, LDL-C, and CIRS score (**Figure 2**). These variables were subsequently incorporated into a multivariate logistic regression model. Multivariate analysis showed that being female [odds ratio (OR): 2.48, 95% CI: 1.50-4.10, p < 0.001], having a lower BMI (OR: 0.92, 95% CI: 0.86-0.98, p = 0.015), lower LDL-C (OR: 0.77, 95% CI: 0.61-0.98, p = 0.031), and higher CIRS score (OR: 1.11, 95% CI: 1.05-1.18, p < 0.001) were independent associated with the risk of depression (**Table 2**).

**Table 2:** Multivariate logistic regression for depression predictors.

Variables	OR (95%CI)	*p*-value
Sex (%)		
male	1.00 (Reference)	
female	2.48 (1.50,4.10)	< 0.001
BMI (kg/m^2^)	0.92 (0.86,0.98)	0.015
LDL-C (mmol/L)	0.77 (0.61,0.98)	0.031
CIRS score	1.11 (1.05,1.18)	< 0.001

Model was adjusted by sex, educational level, BMI, TC, LDL-C, and CIRS score as screened in LASSO regressions. OR, Odds Ratio; CI, Confidence Interval.

To explore the relationship between CIRS scores and depression, we analyzed the associations across different subgroups based on sex, age, duration of DM, smoking history, and drinking history. Interestingly, the CIRS score exhibited a more robust association with depression in certain populations. Among the different groups, the relationship between the CIRS score and depression was statistically significant in the following subgroups: males (OR:1.14, 95% CI: 1.06-1.22, p < 0.001), individuals under 60 years of age (OR: 1.16, 95%CI: 1.05-1.28, p = 0.004), those with a DM duration less than 5 years (OR: 1.24, 95% CI: 1.07-1.42, p = 0.003), individuals with no smoking history (OR:1.12, 95% CI: 1.02-1.24, p = 0.018), and those without a history of alcohol use (OR: 1.15, 95% CI: 1.05-1.25, p = 0.003). In contrast, the association was weaker and not statistically significant in females (OR: 1.08, 95% CI: 0.98-1.19, p = 0.141), those aged 60 years or older (OR: 1.07, 95% CI: 0.98-1.16, p = 0.123), individuals with diabetes for 5 years or longer (OR: 1.04, 95% CI: 0.97-1.12, p = 0.295), and those with a history of smoking (OR: 1.10, 95% CI: 1.01-1.21, p = 0.038), or alcohol use (OR: 1.09, 95% CI: 0.98-1.22, p = 0.094) (**Table 3**).

**Table 3:** Association between CIRS score and depression in different populations.

Variables		OR (95%CI)	*p*-value
Sex	Male	1.14 (1.06, 1.22)	< 0.001*
	Female	1.08 (0.98, 1.19)	0.141
Age	< 60	1.16 (1.05, 1.28)	0.004*
	≥ 60	1.07 (0.98, 1.16)	0.123
DM duration	< 5	1.24 (1.07, 1.42)	0.003*
	≥ 5	1.04 (0.97, 1.12)	0.295
Smoking	No	1.12 (1.02, 1.24)	0.018*
	Yes	1.10 (1.01, 1.21)	0.038*
Drinking	No	1.15 (1.05, 1.25)	0.003*
	Yes	1.09 (0.98, 1.22)	0.094

OR, Odds Ratio; CI, Confidence Interval, *p < 0.05.

**Figure 3:**
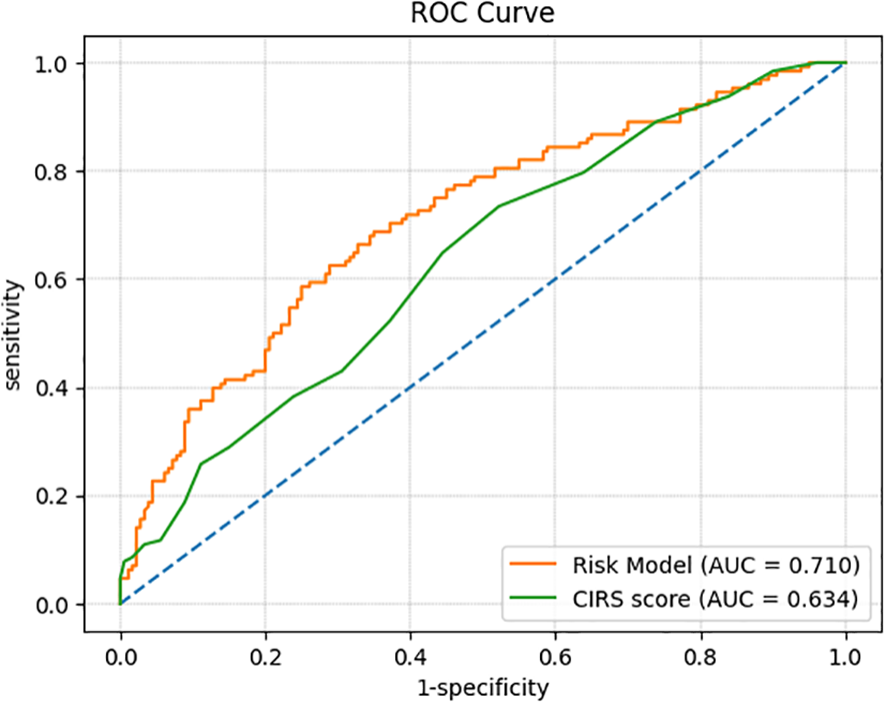
Curves of receiver operating characteristic.

Subsequently, we performed a ROC analysis to assess the predictive capacity of the CIRS score for depression. The analysis revealed an AUC of 0.634 (95%CI: 0.572-0.696, p < 0.001), with an optimal cutoff value of 8.5, yielding a sensitivity of 0.734 and specificity of 0.478. In multivariate analysis, we integrated additional variables, including sex, educational level, BMI, TC, LDL-C, and CIRS score, to construct a predictive model. The AUC of this model was 0.710 (95% CI: 0.651-0.769, p < 0.001), with a cutoff value of 0.404, resulting in a sensitivity of 0.688 and specificity of 0.650. (**Table 4**; **Figure 3**).

**Table 4:** Receiver operating characteristic model and the predictive values.

Characteristics	AUC	95%CI	*p*- value	Cut-off Value	Sensitivity	Specificity
CIRS score	0.634	0.572, 0.696	< 0.001*	8.500	0.734	0.478
Risk Model	0.710	0.651, 0.769	< 0.001*	0.404	0.688	0.650

Model was adjusted by gender, educational level, BMI, TC, LDL-C, and CIRS score with *p* < 0.05 in multivariate analysis. AUC, Area Under Curve; CI, confidence interval; *p < 0.05.

## 4 Discussion

This study provides important insights into the risk factors for depression in patients with type 2 diabetes. Our main findings include: (1) female sex, lower BMI, lower LDL-C levels and higher CIRS score were identified as independent risk factors for depression in T2DM patients; (2) CIRS scores showed a more significant association with depression in specific subgroups (men, age < 60 years, DM duration < 5 years, and no smoking or drinking history); (3) A predictive model incorporating sex, BMI, LDL-C and CIRS score demonstrated high accuracy in assessing depression risk.

Our study found that female sex was independently associated with a higher risk of depression, a finding consistent with numerous studies (23–25). Research has consistently showed that women with T2DM experience higher rates of depression compared to men, with one study indicating that the prevalence of depression among women was significantly higher (17.1% vs. 9.3%) than in men with diabetes ability could be due to a combination of hormonal, psychosocial, and genetic factors, although further investigation into the underlying mechanisms is needed (26). Our findings (OR=2.48, 95% CI: 1.50-4.10, p < 0.001) confirm that female sex is an independent risk factor for depression in T2DM, aligning with previous research and highlighting the need for gender-specific strategies in the management of diabetes and associated depression.

Previous studies have produced mixed results regarding the association between BMI and depression. Some studies suggested that individuals with high BMI are more prone to developing depression (27–30), a relationship often explained by factors such as physical inactivity, poorer quality of life, and increased vulnerability to social stigma, all of which are commonly observed in obese individuals (31). In contrast, other studies have reported a negative correlation between BMI and depression, with underweight individuals also showing a heightened risk for depressive symptoms (32). In addition, some studies have found no significant relationship between BMI and depression, but they cannot rule out the association between them (33). However, recent literature increasingly supports a non-linear, U-shaped association between BMI and depression. Both high and low BMI extremes appear to contribute to an elevated risk of depression (34–36). A pivotal study identified a BMI inflection point at approximately 25.719 kg/m2, suggesting that the lowest depression risk occurs within the World Health Organization’s defined healthy BMI range (18.5 ≦ BMI < 25.0) (37). This discrepancy in findings across studies may stem from variations in sample size, population characteristics, and study design, as some studies may not have adequately accounted for underweight individuals or focused exclusively on specific cohorts, such as the elderly, students, or middle-aged individuals (38, 39). Our findings support this U-shaped relationship, with a negative association between BMI and depression observed in our type 2 diabetes cohort (OR=0.92, 95% CI: 0.86-0.98, p=0.015). While our study population was limited to individuals with type 2 diabetes, the data are consistent with the broader literature, indicating that both low and high BMI are associated with an increased risk of depression. Specifically, our study suggests that low BMI may also serve as an independent risk factor for depression in this group, reinforcing the need for a nuanced approach to weight management in the clinical setting. Clinically, these findings highlight the importance of maintaining a balanced and healthy body weight. Excessive pursuit of weight loss, particularly at the expense of nutritional or psychological well-being, may not only fail to improve mental health but could, in fact, exacerbate depressive symptoms. Therefore, clinicians should prioritize a holistic approach to managing both physical and mental health, advocating for regular physical activity, balanced nutrition, and fostering a positive body image. These strategies are essential not only for managing type 2 diabetes but also for promoting overall mental health in this population.

In contrast to previous studies that have linked higher LDL-C levels with an increased risk of depression in patients with type 2 diabetes (40), our findings indicate that lower LDL-C levels are more likely to be associated with depression (OR=0.77, 95% CI: 0.61-0.98, p=0.031). This discrepancy may be partially explained by the influence of lipid-lowering drug adherence. Previous research has shown that individuals with poor adherence medication tend to have elevated LDL-C levels, which might explain the higher observed LDL-C levels in depressed patients compared to the normal those in the general population (41). In contrast, patients with better adherence may exhibit lower LDL-C levels, potentially contributing to the divergent findings in the literature. Furthermore, the relationship between depression and LDL-C is complex and involves intricate biological mechanisms. Cholesterol, transported by LDL-C particles in peripheral circulation, is also synthesized within the brain (42). Lower peripheral LDL-C levels could reflect a depletion of cholesterol in the brain, which may impair neuronal function. A reduction in brain cholesterol has been shown to decrease serotonin activity, a key neurotransmitter implicated in depression. Thus, lower LDL-C levels in peripheral systems may be linked to a reduction in serotonin and its receptors, leading to depressive symptoms (43, 44). The association between depression and LDL-C levels is still controversial (45). Some studies reported slightly higher LDL-C levels in depressed patients (46), while others failed to show a positive correlation (47). In addition, several studies have proposed a U-shaped relationship between serum LDL-C and depression, suggesting that both high and low serum LDL-C levels may increase depression risk (48). It is hypothesized that lower LDL-C levels may precede depression onset, but as depressive symptoms progress, patients may engage in behaviors leading to weight gain, ultimately resulting in elevated LDL-C levels. This highlights the dynamic, multifactorial nature of the relationship between LDL-C and depression. It is important to note that since serum samples from patients are only taken once, this may misrepresent the true serum cholesterol before depressive symptoms occur. In addition, detailed statin use data were lacking in our study, which could affect the relationship between depression and LDL-C. But other studies have found that the relationship may not be related to preventative use of cholesterol-lowering drugs (49). Future studies are essential to unravel the complex interplay between LDL-C levels, lipid metabolism, statin use and depression in patients with type 2 diabetes, providing a clearer understanding of their mechanistic links and clinical implications.

In examining the relationship between comorbidities and depression in T2DM, previous studies have generally not addressed the combined effect of multiple comorbid conditions as a whole. To fill this gap, we utilized the CIRS to evaluate the overall burden and severity of comorbidities in this population. Our study demonstrated that higher CIRS scores were independently associated with an increased risk of depression (OR=1.11, 95%CI: 1.05-1.18, p < 0.001), further corroborating the notion that comorbidities contribute significantly to the development of depression in diabetic patients (50). Previous research has shown that comorbidities related to both macrovascular and microvascular complications are important predictors of depression in diabetes (51–53). These findings underscore the critical role that comorbid conditions play in both the prognosis and overall quality of life of individuals with diabetes. The association between CIRS scores and depression can be elucidated through the lens of cytokine mechanisms. Elevated levels of proinflammatory cytokines, which are frequently observed in patients with multiple comorbidities, have the potential to disrupt neurobiological pathways that are crucial for mood regulation. For instance, chronic non-infectious diseases such as diabetes mellitus (54), coronary artery disease (55), hypertension (56), and chronic obstructive pulmonary disease (57) are known to lead to increased levels of cytokines (e.g., interleukin-2, tumor necrosis factor), which can induce depressive symptoms (58, 59). This suggests that the burden of multiple chronic diseases can result in heightened psychological stress, which in turn can precipitate depression. Clinicians can leverage the CIRS score to identify patients who are at a higher risk of developing depression and intervene accordingly (60). In our study, we considered the cumulative effect of these comorbidities and found that, when assessed together, they were a significant independent risk factor for depression in T2DM patients. Further analysis revealed that the association between the CIRS score and depression varied across different subgroups. Specifically, we observed a stronger correlation in men, younger patients (<60 years), those with a shorter duration of diabetes (<5 years), and individuals without a history of smoking or alcohol consumption. These findings suggest that the interplay between comorbidities and depression may be influenced by demographic and clinical factors, and certain subgroups may be more vulnerable to the mental health impacts of comorbidity burden. Further investigation into the CIRS-depression link is crucial to elucidate the role of multi-system comorbidities in the mental health outcomes of T2DM patients.

The ROC analysis revealed that the AUC of the prediction model was 0.710 (95% CI: 0.651–0.769; p < 0.001), indicating a satisfactory level of predictive accuracy. The optimal cut-off value was determined to be 0.404, at which the model achieved a sensitivity of 0.688 and a specificity of 0.650. These results suggest that the model possesses a robust predictive capacity for identifying the occurrence of depression in patients with type 2 diabetes. The predictive power of the single-factor CIRS score was also impressive, with an AUC of 0.634 (95% CI: 0.572-0.696, p < 0.001), and demonstrated moderate predictive value with a sensitivity of 0.734 and a specificity of 0.478 when the optimal cut-off value was 8.5. We developed a predictive model incorporating sex, BMI, LDL-C, and CIRS scores to assess the risk of depression in T2DM patients, providing a robust, evidence-based framework for early identification of mental health risks in this population. This model can aid clinicians in pinpointing diabetic patients at high risk for depression, facilitating timely interventions that could improve both mental health and long-term outcomes.

However, this study has several limitations that should be acknowledged. Firstly, the primary limitation is the sample size. Although we included a substantial number of participants, the sample may not be representative of the broader population. This could potentially limit the generalizability of our findings to other populations or settings. Secondly, as a cross-sectional analysis conducted at a single center, it may be subject to selection bias and may not fully reflect the demographic diversity of broader populations. Future research should include multi-center, prospective studies with larger, more diverse cohorts to validate these results and explore their broader applicability. Thirdly, the reliance on self-reported questionnaires for assessing comorbidities and depression introduces potential reporting bias.

## 5 Conclusion

In conclusion, our study identified several independent risk factors for depression in patients with type 2 diabetes, including female sex, lower BMI, lower LDL-C levels, and higher CIRS scores. We also demonstrated that the CIRS score was particularly effective in predicting depression risk in specific subgroups, including males, individuals under 60 years of age, those with a disease duration of less than 5 years, and those without a history of smoking or alcohol consumption. Additionally, we developed a predictive model incorporating sex, BMI, LDL-C, and CIRS scores, which showed strong performance in assessing depression risk in this population. By evaluating both the traditional and multi-system comorbidities of diabetic patients, this study offers a comprehensive approach to understanding the complex interplay between physical health and mental well-being, providing valuable insights for early identification and intervention in the management of depression in type 2 diabetes. In the future, we may need to emphasize comorbidity and mental health assessment in routine diabetes care.

## Data Availability

The raw data supporting the conclusions of this article will be made available by the authors, without undue reservation.

## References

[1] Ong KL Stafford LK McLaughlin SA Boyko EJ Vollset SE Smith AE. Global, regional, and national burden of diabetes from 1990 to 2021, with projections of prevalence to 2050: a systematic analysis for the Global Burden of Disease Study 2021. Lancet. (2023) 402:203–34. doi: 10.1016/S0140-6736(23)01301-6 PMC10364581 37356446

[2] The L. Diabetes: a defining disease of the 21st century. Lancet. (2023) 401(10394):2087. doi: 10.1016/S0140-6736(23)01296-5 37355279

[3] Aguado A Moratalla-Navarro F López-Simarro F Moreno V. MorbiNet: multimorbidity networks in adult general population. Analysis of type 2 diabetes mellitus comorbidity. Sci Rep. (2020) 10(1):2416. doi: 10.1038/s41598-020-59336-1 32051506 PMC7016191

[4] Teck J. Diabetes-associated comorbidities. Primary Care: Clinics Office Practice. (2022) 49:275–86. doi: 10.1016/j.pop.2021.11.004 35595482

[5] Zghebi SS Steinke DT Rutter MK Ashcroft DM. Eleven-year multimorbidity burden among 637 255 people with and without type 2 diabetes: a population-based study using primary care and linked hospitalisation data. BMJ Open. (2020) 10(7):e033866. doi: 10.1136/bmjopen-2019-033866 PMC7358107 32611677

[6] Kao K-L Sung F-C Tzang R-F Huang H-C Lin C-L Fang C-K. Associations of diabetes severity and risk of depression: a population-based cohort study. J Affect Disord. (2020) 273:476–81. doi: 10.1016/j.jad.2020.04.066 32560943

[7] Liu Y Huang S-Y Liu D-L Zeng X-X Pan X-R Peng J. Bidirectional relationship between diabetes mellitus and depression: Mechanisms and epidemiology. World J Psychiatry. (2024) 14:1429–36. doi: 10.5498/wjp.v14.i10.1429 PMC11514559 39474387

[8] Horsbøl TA Hoffmann SH Thorsted AB Rosenkilde S Lehn SF Kofoed-Enevoldsen A. Diabetic complications and risk of depression and anxiety among adults with type 2 diabetes. Diabetic Med. (2023) 41(4):e15272. doi: 10.1111/dme.15272 38157285

[9] Tran NMH Nguyen QNL Vo TH Le TTA Ngo NH. Depression among patients with type 2 diabetes mellitus: prevalence and associated factors in hue city, Vietnam. Diabetes Metab Syndrome Obesity: Targets Ther. (2021) 14:505–13. doi: 10.2147/DMSO.S289988 PMC7869718 33568927

[10] Khaledi M Haghighatdoost F Feizi A Aminorroaya A. The prevalence of comorbid depression in patients with type 2 diabetes: an updated systematic review and meta-analysis on huge number of observational studies. Acta Diabetologica. (2019) 56:631–50. doi: 10.1007/s00592-019-01295-9 30903433

[11] Koyama AK Hora IA Bullard KM Benoit SR Tang S Cho P. State-specific prevalence of depression among adults with and without diabetes — United states, 2011–2019. Preventing Chronic Disease. (2023) 20:E70. doi: 10.5888/pcd20.220407 37562067 PMC10431924

[12] AbdElmageed RM Mohammed Hussein SM. Risk of depression and suicide in diabetic patients. Cureus. (2022) 14(1):e20860. doi: 10.7759/cureus.20860 35145767 PMC8803388

[13] Almohammadi AM Bawazeer SS Balkhair JJ Rajab AA. Assessment of the relationship between depression and treatment compliance in chronically-ill patients in Jeddah, Saudi Arabia. Trop J Pharm Res. (2021) 20:177–82. doi: 10.4314/tjpr.v20i1.25

[14] Bayani MA Shakiba N Bijani A Moudi S. Depression and quality of life in patients with type 2 diabetes mellitus. Caspian J Internal Med. (2022) 13(2):335–42. doi: 10.22088/cjim.13.2.3 PMC9301220 35919653

[15] Wu C-S Hsu L-Y Pan Y-J Wang S-H. Associations between antidepressant use and advanced diabetes outcomes in patients with depression and diabetes mellitus. J Clin Endocrinol Metab. (2021) 106(12):e5136–46. doi: 10.1210/clinem/dgab443 34259856

[16] Cooper ZW O’Shields J Ali MK Chwastiak L Johnson LCM. Effects of integrated care approaches to address co-occurring depression and diabetes: A systematic review and meta-analysis. Diabetes Care. (2024) 47:2291–304. doi: 10.2337/dc24-1334 PMC12328256 39602589

[17] Benderra MA Serrano AG Paillaud E Tapia CM Cudennec T Chouaïd C. Prognostic value of comorbidities in older patients with cancer: the ELCAPA cohort study. ESMO Open. (2023) 8(5):101831. doi: 10.1016/j.esmoop.2023.101831 37832389 PMC10594025

[18] Zung WWK. A self-rating depression scale. Arch Gen Psychiatry. (1965) 12:63–70. doi: 10.1001/archpsyc.1965.01720310065008 14221692

[19] Esbenshade AJ Lu L Friedman DL Oeffinger KC Armstrong GT Krull KR. Accumulation of chronic disease among survivors of childhood cancer predicts early mortality. J Clin Oncol. (2023) 41:3629–41. doi: 10.1200/JCO.22.02240 PMC10325751 37216619

[20] Geiss R Sebaste L Valter R Poisson J Mebarki S Conti C. Complications and discharge after radical cystectomy for older patients with muscle-invasive bladder cancer: the ELCAPA-27 cohort study. Cancers. (2021) 13(23). doi: 10.3390/cancers13236010 PMC8656698 34885120

[21] Shouse G Kaempf A Yashar D Sigmund AM Smilnak G Bair SM. Impact of comorbidities on outcomes and toxicity in patients treated with CAR T-cell therapy for diffuse large B cell lymphoma (DLBCL): A multicenter rwe study. Blood. (2021) 138:529. doi: 10.1182/blood-2021-149735

[22] Mato AR Roeker LE Lamanna N Allan JN Leslie L Pagel JM. Outcomes of COVID-19 in patients with CLL: a multicenter international experience. Blood. (2020) 136:1134–43. doi: 10.1182/blood.2020006965 PMC7472711 32688395

[23] Majumdar S Sinha B Dastidar BG Gangopadhyay KK Ghoshal S Mukherjee JJ. Assessing prevalence and predictors of depression in Type 2 Diabetes Mellitus (DM) patients – The DEPDIAB study. Diabetes Res Clin Pract. (2021) 178:108980. doi: 10.1016/j.diabres.2021.108980 34329694

[24] Wu D Shi Z Wu C Sun W Jin G. Sex differences in symptom network structure of depression, anxiety, and self-efficacy among people with diabetes: a network analysis. Front Public Health. (2024) 12. doi: 10.3389/fpubh.2024.1368752 PMC10941846 38496386

[25] Ning F Zhang D Xue B Zhang L Zhang J Zhu Z. Synergistic effects of depression and obesity on type 2 diabetes incidence in Chinese adults. J Diabetes. (2019) 12:142–50. doi: 10.1111/1753-0407.12968 31287240

[26] Rajput R Gehlawat P Gehlan D Gupta R Rajput M. Prevalence and predictors of depression and anxiety in patients of diabetes mellitus in a tertiary care center. Indian J Endocrinol Metab. (2016) 20(6):746–51. doi: 10.4103/2230-8210.192924 PMC5105554 27867873

[27] Badillo N Khatib M Kahar P Khanna D. Correlation between body mass index and depression/depression-like symptoms among different genders and races. Cureus. (2022) 14(2):e21841. doi: 10.7759/cureus.21841 35291524 PMC8896404

[28] Eik-Nes TT Tokatlian A Raman J Spirou D Kvaløy K. Depression, anxiety, and psychosocial stressors across BMI classes: A Norwegian population study - The HUNT Study. Front Endocrinol. (2022) 13. doi: 10.3389/fendo.2022.886148 PMC9399822 36034441

[29] Wang F Jia L. BMI moderates the relationship between depression and chronic obstructive pulmonary disease: A cross−sectional survey. Heart Lung. (2024) 68:68–73. doi: 10.1016/j.hrtlng.2024.06.013 38936063

[30] Zhu X Yue Y Li L Zhu L Cai Y Shu Y. The relationship between depression and relative fat mass (RFM): A population-based study. J Affect Disord. (2024) 356:323–8. doi: 10.1016/j.jad.2024.04.031 38614443

[31] Magnusson PKE Rasmussen F Lawlor DA Tynelius P Gunnell D. Association of body mass index with suicide mortality: A prospective cohort study of more than one million men. Am J Epidemiol. (2006) 163:1–8. doi: 10.1093/aje/kwj002 16269577

[32] Qiao Z Wang Z Qiu J Zhang J Cao W. Analysis of the effect of BMI on depression and anxiety among older adults in China: the mediating role of ADL and IADL. Front Public Health. (2024) 12. doi: 10.3389/fpubh.2024.1387550 PMC11408477 39296846

[33] Naz SS Ahmad F. Depression associated with body mass index in adolescent girls in a subset of karachi population. Cureus. (2022) 14(5):e24730. doi: 10.7759/cureus.24730 35686272 PMC9170365

[34] Ma W Yan Z Wu W Li D Zheng S Lyu J. Dose-response association of waist-to-height ratio plus BMI and risk of depression: evidence from the NHANES 05–16. Int J Gen Med. (2021) 14:1283–91. doi: 10.2147/IJGM.S304706 PMC8055360 33883926

[35] Cui H Xiong Y Wang C Ye J Zhao W. The relationship between BMI and depression: a cross-sectional study. Front Psychiatry. (2024) 15. doi: 10.3389/fpsyt.2024.1410782 PMC11534730 39502295

[36] Lee JH Park SK Ryoo JH Oh CM Choi JM McIntyre RS. U-shaped relationship between depression and body mass index in the Korean adults. Eur Psychiatry. (2020) 45:72–80. doi: 10.1016/j.eurpsy.2017.05.025 28738292

[37] Li C Li X Li Y Niu X. The nonlinear relationship between body mass index (BMI) and perceived depression in the chinese population. Psychol Res Behav Manage. (2023) 16:2103–24. doi: 10.2147/PRBM.S411112 PMC10263158 37325255

[38] Noh J-W Kwon YD Park J Kim J. Body mass index and depressive symptoms in middle aged and older adults. BMC Public Health. (2015) 15:310. doi: 10.1186/s12889-015-1663-z 25884564 PMC4383216

[39] Kim T Kim JJ Kim MY Kim SK Roh S Seo JS. A U-shaped association between body mass index and psychological distress on the multiphasic personality inventory: retrospective cross-sectional analysis of 19-year-old men in korea. J Korean Med Sci. (2015) 30(6):793–801. doi: 10.3346/jkms.2015.30.6.793 PMC4444482 26028934

[40] Dehesh T Dehesh P Shojaei S. Prevalence and associated factors of anxiety and depression among patients with type 2 diabetes in kerman, southern Iran. Diabetes Metab Syndrome Obesity: Targets Ther. (2020) 13:1509–17. doi: 10.2147/DMSO.S249385 PMC7211308 32440180

[41] Katzmann J Mahfoud F Böhm M Schulz M Laufs U. Association of medication adherence and depression with the control of low-density lipoprotein cholesterol and blood pressure in patients at high cardiovascular risk. Patient Preference Adherence. (2018) 13:9–19. doi: 10.2147/PPA.S182765 30587940 PMC6302826

[42] Orth M Bellosta S. Cholesterol: its regulation and role in central nervous system disorders. Cholesterol. (2012) 2012:1–19. doi: 10.1155/2012/292598 PMC3483652 23119149

[43] Dreja K Voldstedlund M Vinten J Tranum-Jensen J Hellstrand P Swaürd K. Cholesterol depletion disrupts caveolae and differentially impairs agonist-induced arterial contraction. Arteriosclerosis Thrombosis Vasc Biol. (2002) 22:1267–72. doi: 10.1161/01.ATV.0000023438.32585.A1 12171786

[44] Sommer B Montaño LM Carbajal V Flores-Soto E Ortega A Ramírez-Oseguera R. Extraction of membrane cholesterol disrupts caveolae and impairs serotonergic (5-HT2A) and histaminergic (H1) responses in bovine airway smooth muscle: role of Rho-kinase. Can J Physiol Pharmacol. (2009) 87:180–95. doi: 10.1139/Y08-114 19295659

[45] Ong KL Morris MJ McClelland RL Maniam J Allison MA Rye KA. Lipids, lipoprotein distribution and depressive symptoms: the Multi-Ethnic Study of Atherosclerosis. Trans Psychiatry. (2016) 6:e962–e. doi: 10.1038/tp.2016.232 PMC5290355 27898070

[46] Mehdi SMA Costa AP Svob C Pan L Dartora WJ Talati A. Depression and cognition are associated with lipid dysregulation in both a multigenerational study of depression and the National Health and Nutrition Examination Survey. Trans Psychiatry. (2024) 14(1):142. doi: 10.1038/s41398-024-02847-6 PMC10928164 38467624

[47] Zhong X Ming J Li C. Association between dyslipidemia and depression: a cross-sectional analysis of NHANES data from 2007 to 2018. BMC Psychiatry. (2024) 24(1):893. doi: 10.1186/s12888-024-06359-x 39643888 PMC11622500

[48] Persons JE Fiedorowicz JG. Depression and serum low-density lipoprotein: A systematic review and meta-analysis. J Affect Disord. (2016) 206:55–67. doi: 10.1016/j.jad.2016.07.033 27466743 PMC6201299

[49] Tedders SH Fokong KD McKenzie LE Wesley C Yu L Zhang J. Low cholesterol is associated with depression among US household population. J Affect Disord. (2011) 135:115–21. doi: 10.1016/j.jad.2011.06.045 21802743

[50] de Groot M Anderson R Freedland KE Clouse RE Lustman PJ. Association of depression and diabetes complications: A meta-analysis. Psychosomatic Med. (2001) 63:619–30. doi: 10.1097/00006842-200107000-00015 11485116

[51] Katon W Russo J Lin EHB Heckbert SR Ciechanowski P Ludman EJ. Depression and diabetes: factors associated with major depression at five-year follow-up. Psychosomatics. (2009) 50:570–9. doi: 10.1016/S0033-3182(09)70858-8 PMC3087499 19996227

[52] van Steenbergen-Weijenburg KM van Puffelen AL Horn EK Nuyen J Sytze van Dam P van Benthem TB. More co-morbid depression in patients with Type 2 diabetes with multiple complications. observational study at specialized outpatient clinic. Diabetic Med. (2010) 28:86–9. doi: 10.1111/j.1464-5491.2010.03125.x 21210541

[53] Bruce DG Casey G Davis WA Starkstein SE Clarnette RC Foster JK. Vascular depression in older people with diabetes. Diabetologia. (2006) 49:2828–36. doi: 10.1007/s00125-006-0478-y 17039347

[54] Song S Ni J Sun Y Pu Q Zhang L Yan Q. Association of inflammatory cytokines with type 2 diabetes mellitus and diabetic nephropathy: a bidirectional Mendelian randomization study. Front Med. (2024) 11. doi: 10.3389/fmed.2024.1459752 PMC11580751 39574905

[55] Wang C Liu S Yang Y Kamronbek R Ni S Cheng Y. Interleukin-4 and Interleukin-17 are associated with coronary artery disease. Clin Cardiol. (2023) 47(2):e24188. doi: 10.1002/clc.24188 38146141 PMC10823557

[56] dos Passos RR Santos CV Priviero F Briones AM Tostes RC Webb RC. Immunomodulatory activity of cytokines in hypertension: A vascular perspective. Hypertension. (2024) 81:1411–23. doi: 10.1161/HYPERTENSIONAHA.124.21712 PMC11168883 38686582

[57] Chen J Li X Huang C Lin Y Dai Q. Change of serum inflammatory cytokines levels in patients with chronic obstructive pulmonary disease, pneumonia and lung cancer. Technol Cancer Res Treat. (2020) 19:1533033820951807. doi: 10.1177/1533033820951807 33111646 PMC7607805

[58] Kim Y Pang Y Park H Kim O Lee H. Cytokine associated with severity of depressive symptoms in female nurses in Korea. Front Public Health. (2023) 11. doi: 10.3389/fpubh.2023.1194519 PMC10457120 37637801

[59] Rengasamy M Marsland A McClain L Kovats T Walko T Pan L. Longitudinal relationships of cytokines, depression and anhedonia in depressed adolescents. Brain Behavior Immunity. (2021) 91:74–80. doi: 10.1016/j.bbi.2020.09.004 32919038 PMC7952030

[60] Iosifescu DV Nierenberg AA Alpert JE Smith M Bitran S Dording C. The impact of medical comorbidity on acute treatment in major depressive disorder. Focus. (2005) 3:69–75. doi: 10.1176/foc.3.1.69 14638581

